# Trichomonas Detection in Leucorrhea Based on VIBE Method

**DOI:** 10.1155/2019/5856970

**Published:** 2019-01-14

**Authors:** Xiaohui Du, Lin Liu, Xiangzhou Wang, Jing Zhang, Guangming Ni, Ruqian Hao, Juanxiu Liu, Yong Liu

**Affiliations:** University of Electronic Science and Technology of China, MOEMIL Laboratory, School of Optoelectronic Information, No. 4, Section 2, North Jianshe Road, Chengdu 610054, China

## Abstract

Trichomonas examination is one of the important items in the leucorrhea routine detection. And it cannot be recognized by still images because of the unstable morphology and unfixed focal location caused by motion characteristic. We proposed an improved VIBE algorithm. 6 videos (totally 1414 frames) are collected for testing. In order to compare the effects of the algorithms, we segment each frame artificially as ground truth. Experiments show that percentage of correct classification (PCC) achieves 88%. The proposed improved method can effectively suppress the false detection caused by the formed components such as epithelial cells in the leucorrhea microscopic image and the missed detection caused by the background model update during the movement. At the same time, improvements can effectively suppress smear and ghost areas. The algorithm proposed in this paper can be integrated into the leucorrhea automatic detection system.

## 1. Introduction

**Figure 1:**
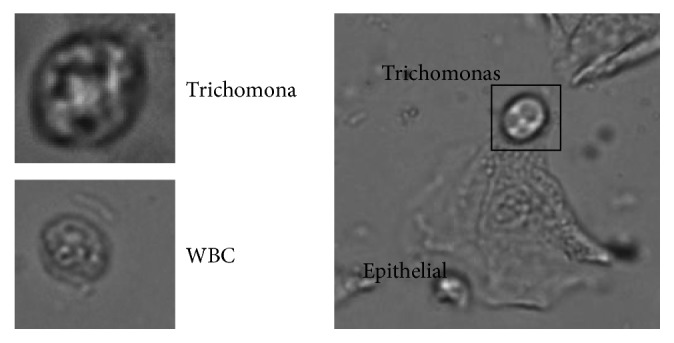
Trichomonas' texture: (a) compared to WBC; (b) trichomonas attached to other cells.

Trichomonas is an important indicator in leucorrhea routine (RT), and the detection of trichomoniasis can prevent a variety of vaginal diseases. At present, the way to detect trichomoniasis in hospitals is under a microscope by manual examination, which is inefficient. So, the microscopic examination algorithm of trichomoniasis is especially necessary for the automation and intelligence of leucorrhea RT. Trichomonas cannot be recognized by still images because of the unstable morphology and unfixed focal location caused by motion characteristic. Trichomonas are morphologically diverse under a microscope. Normally, trichomonas will have flagella and similar texture to white blood cells (WBC) as shown in Figure 1. Its morphological diversity of trichomonas restricts its morphological detection. In most cases, trichomonas may be attached to other cells in the leucorrhea sample, such as epithelial cells, and this diversity poses challenges to ways such as morphologically segmentation.

Because the trichomonas in the fresh leucorrhea sample is still active, it is reflected in the mirror as a motility, and therefore the detection method of the moving target is no doubt a new idea for the detection of the trichomonas.

The frame difference method is the earliest algorithm used to extract the target area of motion, which has strong robustness to the scene containing the moving target. The operation speed is fast, but the method does not generally detect all pixels of the moving object completely, and the phenomenon of “cavity” is often found inside the detected moving object, so the method is applicable to the case of simple motion detection [1]. Yang and Guo [2] and Cheng and Wang [3] proposed the three-frame difference method to solve the vacancy phenomenon of single-frame difference method, but this method has a great influence on lighting and other factors, which is easy to cause error detection.

Background modeling method is the common detection method used in the moving target detection, which uses the median value of pixel value of continuous *K* frame image sequence as the background model. The Gaussian mixture model (GMM) is a background modeling method proposed by Stauffer, which can effectively suppress the false detection caused by background changes and accurately detect the moving target area [4]. However, experiments show that the recognition accuracy of GMM model is low in the trichomonas detection. The kernel density estimation method proposed by Elgammal et al. does not need to make any assumptions about the density distribution of the background, but uses the recent image sample information to accurately estimate pixel points by using the standard kernel function to extract moving objects [5]. The experimental results show that the method has good adaptability in complex outdoor scenes, but the performance to trichomonas detection is not good enough.

VIBE is Barnich's moving target detection method based on pixel points, and it is named the visual background extraction method (VIBE) [6]. This algorithm directly selects a certain number of pixel values randomly according to certain rules for background modeling for each pixel point and then classifies the foreground and background of pixel points using Euclidean distance. The advantage of this algorithm is that it does not need to assume any probability model, and it can detect moving objects in the second frame of video sequence, and the calculation speed is fast. Droogenbroeck and Paquot improved the VIBE algorithm, which improved the PCC (percentages of correct classification) and reduced the PBC (percentage of bad classifications). Although this paper presents a good detection result compared with VIBE, these improvements greatly increase the computation and reduce the real-time performance [7]. Hofmann et al. proposed the pixel-based adaptive segmenter (PBAS) detection method firstly [8]. Based on the advantages of SACON and VIBE, this algorithm combines and improves the detection accuracy of the target further. However, this method is very sensitive to the weak flow of background impurities, resulting in the increase of impurity misdiagnosis.

For the detection of trichomonas, Hao et al. adopted the improved Kalman filtering method to extract trichomonas [9]. Experiments show that this method can adapt to the situation of rapid changes in light. Moreover, when the movement speed of the moving target is slow, or the movement frequency is not high, the accurate extraction of trichomonas cannot be carried out, and the omission ratio is high.

In this paper, we propose an improved VIBE background reconstruction method, with three different background reconstruction optimization methods. Experiments show that PCC achieves 88%, which can filter the misconduct and misdetection caused by impurities effectively. And the phenomena of tailing and ghosts are eliminated. The algorithm proposed in this paper can be integrated into the leucorrhea automatic detection system.

This article is organized as follows. Dataset preparation and the method are described in Section 2.  Section 2.1 is the origin VIBE method and Section 2.2 is the improvement to Section 2.1.  Section 3 introduces the experiment results and the discussion. Conclusions, limitations, and future work are provided in Section 4.

## 2. Dataset Preparation and Method

By collecting a fresh sample, smearing it onto a glass slide, and using a 40x objective lens mounted on a Motic BA210 integrated microscope, we can get the video dataset with a frame rate of 16. All sample collections come from the Fourth Affiliated Hospital of Nanchang University. And all methods were carried out in accordance with relevant guidelines and regulations (the Fourth Affiliated Hospital of Nanchang University regulations). The study has been approved by IRB and other agencies (Ethics Committee of drug clinical trial of the Fourth Affiliated Hospital of Nanchang University, SFYLL-PJ-2015-001), and the samples do not contain any identity and diagnostic information of the subjects. While sampling, doctors would verbally inform the patients. None of the samples are obtained from minor patients or without agreement. For the convenience of comparison, we manually labeled 6 video images, a total of 1414 frames for analysis (ground truth).

### 2.1. VIBE Algorithm

**Figure 2:**
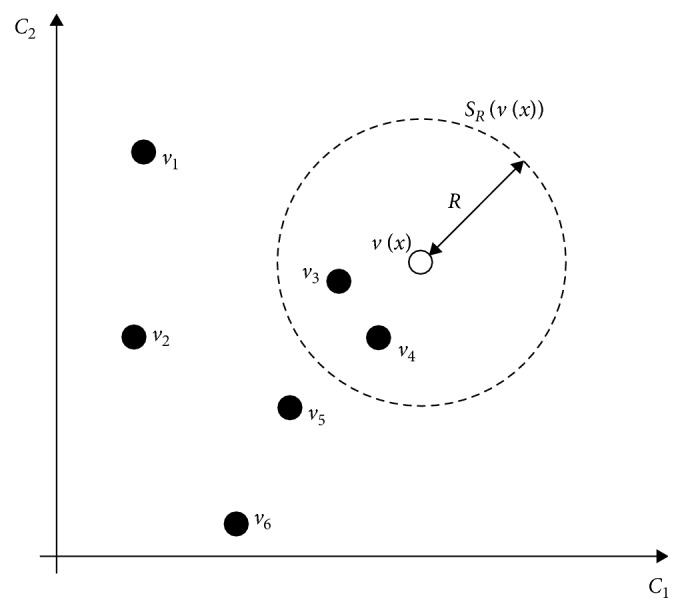
The distribution of pixel value *v*(*x*) and *N* sampling values in the background model in two-dimensional color space.

The background point in VIBE is the pixels that do not move, which should be segmented as 0, while the foreground point is the moving pixels, such as trichomonas in leucorrhea images, which should be segmented as 255. In the VIBE model, a background model is created for each pixel in the first frame. In each frame image, calculate the similarity between the pixels to be classified (foreground and background) and the background model. If the pixel value of a point belongs to the background point, it should be close to the sampling value of the background model sample set. The calculation method of similarity degree is the Euclidean distance between the pixel value *v*(*x*) and the *N* sample values (*v*_1_, *v*_2_,…, *v*_6_) in the background model, which is less than the number of given threshold value (*R*) as shown in Figure 2.

*S*_*R*_(*v*(*x*)) is a color spatial sphere with *v*(*x*) as the center and radius *R* as the threshold. The background model with *N* sample values created for each pixel point *v*(*x*) is as follows:

(1) $$\begin{array}{c} M\left(x\right)=\left\{v_{1},v_{2},\ldots ,v_{N}\right\}. \end{array}$$

Define *U* as the intersection of the background model *M*(*x*) and *S*_*R*_(*v*(*x*)), *v*(*x*) should be classified as background, if *v*(*x*) satisfies the following condition:

(2) $$\begin{array}{c} \#U=\#\left\{S_{R}\left(v\left(x\right)\right)\;\cap \;M\left(x\right)\right\}\geq {\#}_{\text{min}}, \end{array}$$

where # is the counting function and #*U* is the number of samples contained in the intersection *U*. #_min_ is the threshold for whether it is a background or not. The sensitivity of the model is expressed as

(3) $$\begin{array}{c} \frac{{\#}_{\text{min}}}{N}. \end{array}$$

When initializing, VIBE initializes the background model *M*(*x*) by means of single-frame initialization. The specific method is to select *N* pixel values in the current pixel neighborhood randomly:

(4) $$\begin{array}{c} M^{\left(0\right)}=\left\{\left.v^{\left(0\right)}\left(y\right)\right|y\in N_{G}\left(x\right)\right\}. \end{array}$$

The background update of VIBE is random update. According to the update strategy of VIBE, the probability of a sample between time *t* and *t*+*dt* is (*N* − 1)/*N*. Assuming that time is continuous and there is no memory loss during the selection of background, the probability can be

(5) $$\begin{array}{c} P\left(t,t+dt\right)=e^{-\ln\left(N/\left(N-1\right)\right)dt}. \end{array}$$

In the above equation, the sample life cycle in the background model is exponential decay, and the probability of sample retention in time *t*(*t*+*dt*) is independent of time *t*. VIBE uses secondary random sampling to realize the possibility of representing an infinite time window with finite samples. The basic idea is to reduce the frequency of background update and extend the life of samples in the background model. According to the principle that neighborhood pixels have similar temporary distributions, VIBE propagates background samples in the neighborhood. This neighborhood propagation mechanism ensures the spatial consistency of the algorithm and can effectively recover the background area covered by the foreground.

### 2.2. VIBE Improvement

#### 2.2.1. Update of VIBE Background Model

The VIBE model uses three main update strategies for background updates:

- The first is the memoryless update, that is, if a pixel *x* is classified as a background, the algorithm randomly takes a point *v* in the background model *M*(*x*) and updates the value of *v* with the pixel value *p*(*x*) of *x*. This strategy can reduce the probability that the model's value of the background model *v* will be discarded during the update process.
- The second is the time subsampling of the model. The original background point of the model may be background points in consecutive multiple frames; it will reduce the efficiency of the operation if the background model is updated every frame. VIBE uses a random update strategy (adjust *φ*) to determine whether the background point is updated.
- The third is the update of the spatial domain, which can ensure that video does not produce extensive area error detection in jitter. The specific method is that the field set of the background point *x* is *N*(*x*), *u* is randomly taken from *N*(*x*), and the value of the background model *M*(*u*) of *u* is updated to *p*(*x*). This dissemination update mechanism also determines whether the domain points are updated with a certain probability.

The movement of trichomonas in the video is slow, and there is no significant trajectory. When the model is being updated, we adopt the update strategy with memoryless update and time subsampling. For the update of the neighborhood, we update the spatial neighborhood for the background point *x* on the image of each frame and discard the original dissemination mechanism according to the probability. This is because there are a large number of morphological components such as epithelial cells, bacilli, and cocci in the leucorrhea sample. Since the fresh leucorrhea samples are still active, these morphological components in the video will do a small amount of swimming. Therefore, for each background point *x*, there will be a high probability of swimming. If there is a certain probability to determine whether the field is updated, many background points on morphological components will be falsely detected as the foreground points.

In this paper, we update the domain point in an absolute update method, that is, if one sample point *x* is detected as the background point, then background point model *M*(*y*|*y* ∈ *N*(*x*)) of the field *N*_*G*_(*x*) is fixedly updated. Randomly take a domain background point *y* and randomly update its background model to *v*_*y*_:

(6) $$\begin{array}{c} \left\{\begin{array}{c} v_{y}=p\left(x\right),\\ v_{y}\in M\left(y\;|\;y\in N_{G}\left(x\right)\right). \end{array}\right. \end{array}$$

The probability that the neighborhood point *y* is selected is 1/*N*, and the probability that the neighborhood point retains its background model is (*N* − 1)/*N*. With the continuation of time, the probability that the neighborhood point background model is not updated during *t* + *dt* can be written as

(7) $$\begin{array}{c} P_{N}\left(t,t+dt\right)=e^{-\ln\left(-N/\left(N-1\right)\right)dt}. \end{array}$$

In summary, a point *x* is detected as a background point, and then there are two main situations in which the background model is updated: one is to update *M*(*x*) with a probability of 1/*φ* during time subsampling. The other one is that the neighborhood of the point *x* has a background point *y*. While performing the update calculation, point *x* updates *M*(*x*) with a probability of 1/8, assuming that the number of neighborhood of *y* is 8:

(8) $$\begin{array}{c} \left\{\begin{array}{c} v_{x}=p\left(x\right),\;v_{x}\in M\left(x\right),P=\frac{1}{\phi },\\ v_{x}=p\left(y\right),\;x\in N_{\text{G}}\left(y\right),y\text{ is background},P=\frac{1}{8}. \end{array}\right. \end{array}$$

#### 2.2.2. Update of VIBE Neighborhood Background Model

It is mentioned in Section 2.2.1 that when the point *x* is detected as a background point, the background model of the neighborhood point will be updated. According to the principle of VIBE, when the point *y* is the foreground point, there is no need to update the background model of *y*. Therefore, it is considered that when the neighborhood point *y* of *x* is the foreground point at this time, the background model of *y* does not need to be updated. In judging whether *y* is a foreground point, the method of Section 2.2.1 will undoubtedly introduce more calculations. In order to simplify the foreground and background judgment of *y*, this paper proposes a simplified discriminant method.

Since the motion in the leucorrhea sample video changes slowly, it is considered to introduce a frame difference method to prefetch the foreground. The algorithm flow is as follows:

(1) Extract the foreground image by using the frame difference method:
  (9) $$\begin{array}{c} {\text{Mask}}_{K}=\left|I_{K}-I_{K-1}\right|, \end{array}$$
  where *I*_*K*_ is the *K*th frame, that is, the current frame image, while the *I*_*K*−1_ is the previous frame image. Mask_*K*_ is the absolute value of the difference of the current frame image minus the previous frame image.
(2) Binarize Mask_*K*_ with the Otsu threshold method to obtain Mask_seg_*K*_.
(3) Perform morphological hole filling on Mask_seg_*K*_.
(4) Perform close operation to the Mask_seg_*K*_ with a circular structural unit with size 11 to connect the discrete binary points of the same component:
  (10) $$\begin{array}{c} \text{dilate}\left(f\left(x,y\right),\text{se}\right)=\text{max}\left\{\left.f\left(x-x^{\prime },y-y^{\prime }\right)-se\left(x^{\prime },y^{\prime }\right)\right|\left(x^{\prime },y^{\prime }\right)\in \text{se}\right\},\\ \text{erode}\left(f\left(x,y\right),\text{se}\right)=\text{max}\left\{\left.f\left(x-x^{\prime },y-y^{\prime }\right)-se\left(x^{\prime },y^{\prime }\right)\right|\left(x^{\prime },y^{\prime }\right)\in \text{se}\right\},\\ \text{Mask}\_{\text{seg}}_{K}\left(x,y\right)=\text{erode}\left(\text{dilate}\left(\text{Mask}\_{\text{seg}}_{K}\left(x,y\right)\right)\right), \end{array}$$
  where se is the circular structural unit.
(5) Perform open operation to the Mask_seg_*K*_ with a circular structural unit with size 9 to filter the weak flowing impurities:
  (11) $$\begin{array}{c} \text{Mask}\_{\text{seg}}_{K}\left(x,y\right)=\text{dilate}\left(\text{erode}\left(\text{Mask}\_{\text{seg}}_{K}\left(x,y\right)\right)\right). \end{array}$$

**Figure 3:**
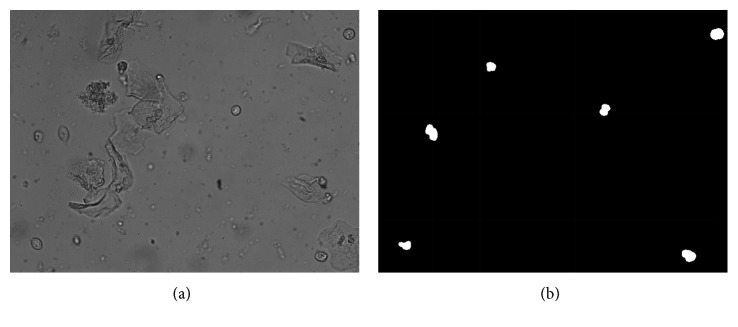
The foreground image obtained by the frame difference method: (a) the original image *I* and (b) the foreground extraction picture Mask_seg_*K*_.

The resulting effect picture Mask_seg is shown in Figure 3.

If the point *x* is the background point, it is first determined whether the neighborhood point *y* is the background when updating the neighborhood background model, that is, whether the corresponding pixel point in Figure 3(b) is 0. The update strategy becomes a random selection of the background neighborhood points of *x* for updating

(12) $$\begin{array}{c} \left\{\begin{array}{c} v_{y}=p\left(x\right),\\ v_{y}\in M\left(y\;|\;y\in N_{\text{G}}\left(x\right),\text{Mask}\_{\text{seg}}_{K}\left(y\right)=0\right). \end{array}\right. \end{array}$$

For the background point *x*, the probability that the neighborhood *y* will be updated is

(13) $$\begin{array}{c} P=\frac{1}{C_{y}},\;\text{if }y\in N_{\text{G}}\left(x\right)\text{ and}\;\text{Mask}\_{\text{seg}}_{K}\left(y\right)=0, \end{array}$$

where *C*_*y*_ is the number of neighborhoods that satisfy the condition, that is, *y* in Mask_seg_*K*_ is the background point (pixel value is 0).

#### 2.2.3. Impurity Filtration

We believe that the point should be a background point if a region is detected as a foreground spot for consecutive *K* frames. Through this improved method, most of the formed components regions can be filtered. This is because the components such as epithelial cells are basically static except for the trichomonas. Although they are affected by the water flow, these impurities undergo a slight movement. If a pixel is detected as a foreground point by successive *K* frames, then we can force it to be a background point, as shown below:

(14) $$\begin{array}{c} {\text{cnt}}_{i+1}\left(x\right)={\text{cnt}}_{i}\left(x\right)+1,\\ m_{i+1}\left(x\right)=0,\;\text{if }{\text{cnt}}_{i+1}\left(x\right)>K, \end{array}$$

where cnt_*i*_(*x*) indicates the cumulative number of times the point *x* is detected as the foreground point at the *i*th frame and *m*(*x*) is the pixel value of foreground image at the *x* position.

In addition, we believe that the foreground point will not appear out of thin air, and the trichomonas must have existed in the field of vision or moved from outside of the vision to the field of vision. Therefore, if a frame of a pixel point *x* is detected as a background, the next frame is a foreground point, and there is no foreground point in the neighborhood of the previous frame of *x*, and then *x* is considered to appear in the field of view out of thin air as follows:

(15) $$\begin{array}{c} m_{i+1}\left(x\right)=0,\;\text{if }m_{i}\left(x\right)=0\;\;\mathrm{and}\;\;m_{i}\left(y\right)=0,y\in N_{G}\left(x\right). \end{array}$$

### 2.3. Main-Process Stream

**Figure 4:**
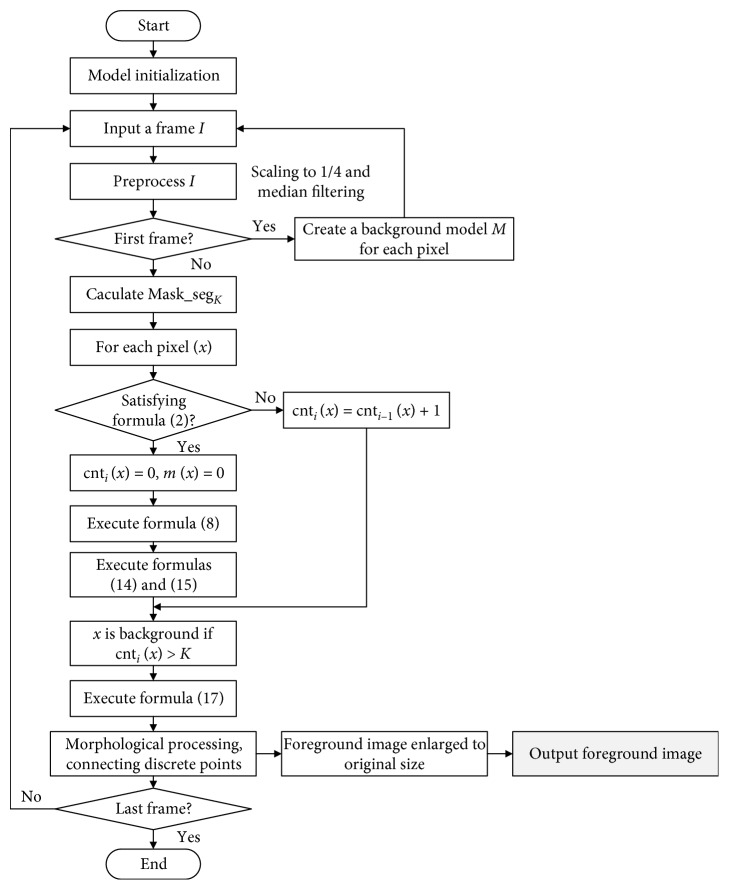
The flow chart of the improved VIBE method.

According to the improved algorithm based on VIBE, the flow chart of total trichomonas detection is shown in Figure 4.

During initialization, *N* = 20, #_min_ is 2, the radius *R* is 40 px, and the subsampling probability *φ* is 3.

## 3. Experimental Results and Discussion

### 3.1. Update of VIBE Background Model

The background model update strategy of Section 2.2.1 can effectively filter out regional misdetection caused by weak sway.

**Figure 5:**
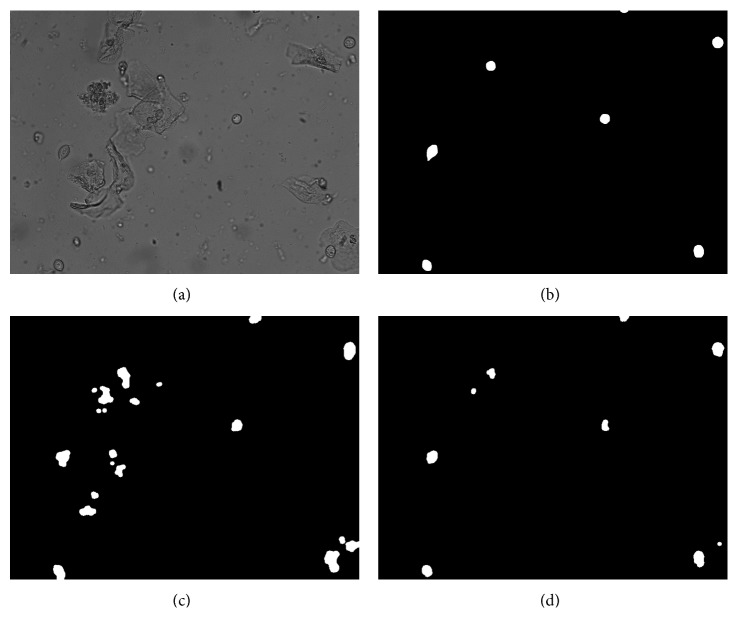
Result of background model update improvement: (a) original image; (b) standard trichomoniasis foreground map; (c) VIBE original algorithm foreground map; (d) improved algorithm (Section 2.2.1) foreground map.

As shown in Figure 5, the 27th frame image in the video image and its foreground segmentation are compared. After the update strategy for the neighborhood point is changed to absolute update, it can effectively suppress foreground segmentation misdetection caused by weak disturbances such as epithelial cells, and the updated foreground effect map is closer to the standard foreground image. Experiments have shown that this improved strategy can increase the percentage of correct classification (PCC) by 10%.

### 3.2. Update of VIBE Neighborhood Background Model

**Figure 6:**
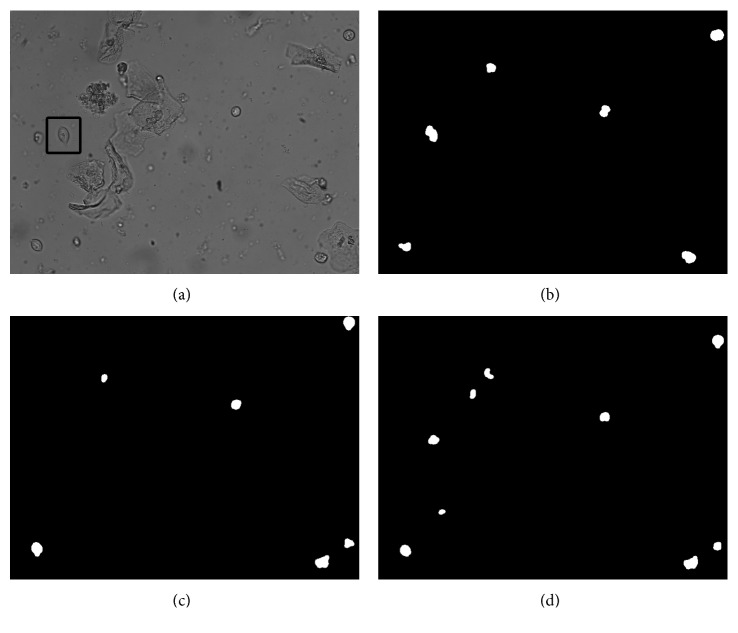
Image effect of background model update improvement: (a) original image; (b) standard trichomoniasis foreground map; (c) VIBE original algorithm foreground map; (d) improved algorithm (Section 2.2.2) foreground map of this paper.

Only when the neighborhood *y* of the background point *x* is the background point, *y* is possible to be updated. This strategy can effectively avoid some of the foreground point *x*′ to be updated as a background point in the next frame detection, due to the background model update of the neighborhood *y*′ as shown in Figure 6.

In Figure 6, the 45th frame in the video image was used for comparison. It was found that the trichomonas at the box in the original VIBE model produced a missed detection, and after the introduction of the strategy (Section 2.2.2) improvement, the phenomenon was improved. Experiments show that this strategy can increase the detection PCC by about 8%.

### 3.3. Impurity Filtration Test

Misdetection of the foreground point can be effectively improved by the two strategies in Section 2.2.3. If pixel consecutive *K* frames are detected as a foreground point, make it as the background point. 

**Figure 7:**
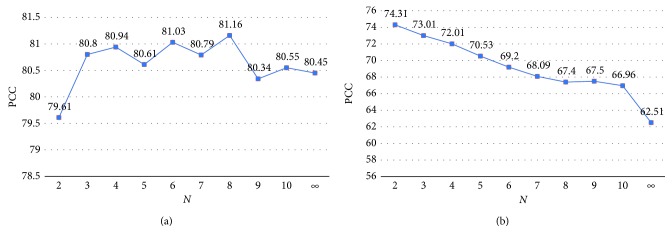
The impact of changes in *K* on PCC with (a) and without (b) strategy in Section 2.2.1.

As shown in Figure 7, the effect of filtering the formed component can be effectively achieved, and the change of *K* has little effect on the final PCC change with the strategy in Section 2.2.1. The average value of the calculated PCC reached more than 80%. In contrast, in the absence of the strategy described in Section 2.2.1, the average value of PCC is smaller, only about 69%, and with the increase of *K*, PCC decreases, reaching the best when *K* takes the minimum value of 2.

**Figure 8:**
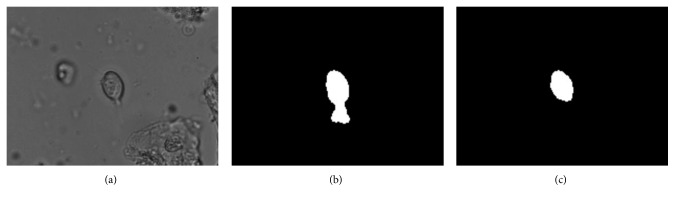
The effect of the strategy in Section 2.2.3 on eliminating the ghost region: (a) the original image; (b) *K* takes infinity; (c) *K* takes 2.

In addition, the introduction of the strategy described in Section 2.2.3 can effectively eliminate the ghost area, as shown in Figure 8.

### 3.4. Comprehensive Detection Effect

Many metrics can be used to assess the output of a background subtraction algorithm given a series of ground-truth segmentation maps. Barnich and Vanogenbroeck [6] use the percentage of correct classification (PCC) to evaluate

(16) $$\begin{array}{c} \text{PCC}=\frac{\text{TP}+\text{TN}}{\text{TP}+\text{TN}+\text{FP}+\text{FN}}, \end{array}$$

where TP accounts for the number of correctly detected foreground pixels; FP accounts for the number of background pixels incorrectly classified as foreground; TN accounts for the number of correctly classified background pixels; and FN accounts for the number of foreground pixels incorrectly classified as background.

However, the number of trichomonas in the leucorrhea sample image is generally small, and the area of the whole map is small. Therefore, the introduction of *TN* in PCC, the black background, will be the majority, so we have changed the PCC as the comparison of PCC is meaningless:

(17) $$\begin{array}{c} \text{PCC}=\frac{\text{TP}}{\text{TP}+\text{FP}+\text{FN}}. \end{array}$$

**Figure 9:**
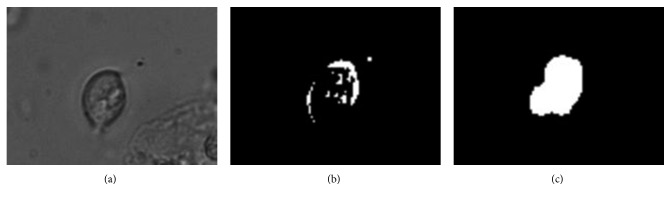
Foreground map discrete points filling and connection: (a) original image; (b) foreground image; (c) processing result map.

In addition, since the foreground image obtained by segmentation is usually composed of discrete points, in order to detect the target such as trichomoniasis, we connect and fill the points with similar distances; the specific methods are hole filling, closing operation, and opening operation as shown in Figure 9.

The final calculation results are as follows.

**Figure 10:**
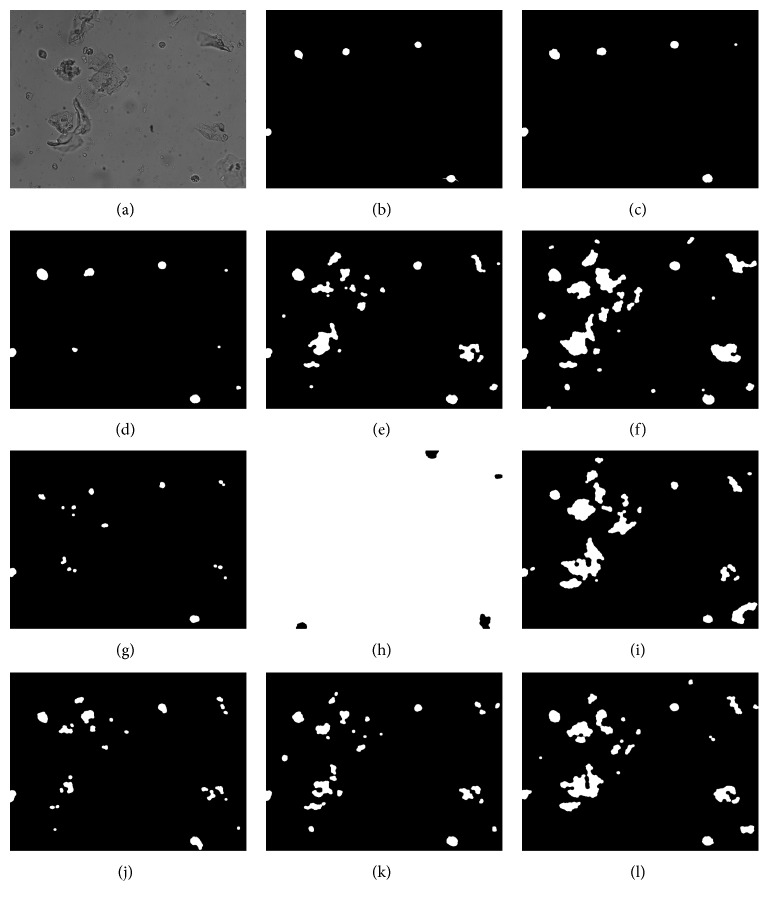
Segmentation foreground renderings: (a) original image; (b) standard foreground image (ground truth); (c) algorithm in this paper; (d) VIBE; (e) frame difference; (f) three-frame difference; (g) GMM; (h) KDE; (i) CodeBook; (j) Kalman; (k) improved Kalman; (l) PBAS.

As can be seen from Table 1, the improved algorithm proposed in this paper has improved by nearly 17% on the original basis. In addition, the effect of the frame difference method and the three-frame difference method is also good because the video shooting conditions are stable, and the test found that when the microscope focal length changes slightly, the effect is very poor, as shown in Figure 10.

The split effect of frame 157 is shown in Figure 10.

In addition, we presented the intersection of union precision (IoU precision) indicator, which was used to determine the detection effect in target detection:

(18) $$\begin{array}{c} \text{IoU}=\frac{A\left(R_{\text{p}}\;\cap \;R_{\text{gt}}\right)}{A\left(R_{\text{p}}\;\cup \;R_{\text{gt}}\right)},\\ \text{IoU}\_\text{precision}=\frac{\text{TP}}{\text{TP}+\text{FP}}, \end{array}$$

where *R*_p_ ∩ *R*_gt_ represents the intersection of predicted box and ground-truth box. And *R*_p_  ∪  *R*_gt_ represents the union. *A* is the function of Area. TP represents the number of bounding box whose IoU is greater than the threshold. FP represents the number of bounding boxes that were mistakenly segmented into foreground targets.

The IoU threshold, which is set to 0.5, and the IoU precision are shown in Table 2.

As can be seen from Table 2, the proposed algorithm has an IoU precision of 94.8%, which is much higher than other foreground extraction algorithms. This also indicates that the algorithm has a good effect on trichomonas detection in leucorrhea.

The algorithm uses the Intel Core (TM) i7-4810MQ 2.80 GHz PC for detection. The average detection time is 98.08 ms, which meets the requirements of real-time video streaming trichomoniasis detection.

## 4. Conclusion

In this paper, a detection algorithm of trichomoniasis in leucorrhea microscopy image based on VIBE algorithm is proposed. The experimental results show that the improved algorithm can effectively extract the trichomoniasis region, and the PCC reaches 88%, which is higher than other video motions algorithm.

The proposed improved method can effectively suppress the false detection caused by the formed components such as epithelial cells in the leucorrhea microscopic image and the missed detection caused by the background model update during the movement. At the same time, improvements can effectively suppress smear and ghost areas. The algorithm has good robustness and strong adaptability, and the recognition effect is good under the weak flow of impurities and weak zoom in the video.

## Figures and Tables

**Table 1:** PCC comparison of different moving target foreground extraction models.

Method	PCC
VIBE [6]	0.7119
Frame difference [1]	0.7557
Three-frame difference [2]	0.7101
GMM [4]	0.3587
KDE [5]	0.0059
CodeBook [10]	0.1336
Kalman [11]	0.3958
Improved Kalman [9]	0.7169
PBAS [8]	0.3087
This paper	0.8803

**Table 2:** Comparison of IoU precision for different moving target foreground extraction models.

Method	IoU precision
VIBE	0.6618
Frame difference	0.8097
Three-frame difference	0.8194
GMM	0.5105
KDE	0.0017
CodeBook	0.2081
Kalman	0.4536
Improved Kalman	0.8554
PBAS	0.3336
This paper	0.9485

## Data Availability

The video of trichomonas data used to support the findings of this study is available from the corresponding author upon request.
